# Electronic and Spintronic Properties of Armchair MoSi_2_N_4_ Nanoribbons Doped by 3D Transition Metals

**DOI:** 10.3390/nano13040676

**Published:** 2023-02-09

**Authors:** Xiao-Qian Su, Xue-Feng Wang

**Affiliations:** 1 Jiangsu Key Laboratory of Thin Films, Institute of Theoretical and Applied Physics, School of Physical Science and Technology, Soochow University, 1 Shizi Street, Suzhou 215006, China; 2 Suzhou Fusong Intelligent Technology Co., Ltd., 415B-959 Jiayuan Rd., Suzhou 215100, China

**Keywords:** TM-aMoSiNNRs, negative differential resistance, rectification effect, spin polarization, diversified functional

## Abstract

Structural and physical properties of armchair MoSi_2_N_4_ nanoribbons substitutionally doped by 3d transition metals (TM) at Mo sites are investigated using the density functional theory combined with the non-equilibrium Green’s function method. TM doping can convert the nonmagnetic direct semiconductor into device materials of a broad variety, including indirect semiconductors, half semiconductors, metals, and half metals. Furthermore the 100% spin filtering behavior in spin-up and spin-down half metals, a negative differential resistance with peak-to-valley ratio over 140 and a rectification effect with ratio over 130 are predicted, as well as semiconductor behavior with high spin polarization.

## 1. Introduction

In the last decade, two-dimensional (2D) materials have been attracting exponentially increasing theoretical and experimental interests in their application on nanodevices for electronics and spintronics [1,2,3,4,5]. Diversified device functions and unique performances have been explored and identified in different 2D materials [6]. It is predicted that 2D materials might replace silicon on the way to continuing Moor’s law for integrated circuits in the sub-nano scale [5]. However, combining devices of different functions into an integrated circuit can be challenged due to the difficulty in matching structures of different materials. In addition, a 2D monolayer material may have its intrinsic imperfections. For example, monolayer phosphorene is unstable upon exposure to air [7], monolayer MoS_2_ has quite low carrier mobility [8], and germanene can be easily oxidized in air [9]. Thus, it is in demand to find a desirable 2D material in which primary device functions can be realized via functionalization.

Recently, the monolayer MoSi_2_N_4_ (MoSiN) has been successfully synthesized by the economic chemical vapor deposition (CVD) method [10]. It has appropriate band gap (1.94 eV), high strength and good environmental stability, which are very favorable for high-performance devices. This has also opened a milestone for the development of 2D materials since it is the first fabricated 2D material without a layered 3D parent. Thus, it cannot be obtained by stripping a massive parent as the previous 2D materials do. The monolayer WSi_2_N_4_ (WSiN) has been synthesized based on the same method, followed by a new class of 2D materials with the molecular formula MA_2_Z_4_. Here, M is a metal element (Mo, W, V, Nb, Ta, Ti, Zr, Hf or Cr), A represents Si or Ge, and Z represents N, P or As. Due to the diversity and adjustability of elements, MA_2_Z_4_ shows very rich physical properties and may be a metal, semi-metal, semiconductor, or magnetic material [10]. For the monolayer MoSiN, the band gap can be tuned through strain engineering [11,12]. Together with other functionalizations such as vacancy, doping, and atomic adsorption, MoSiN is expected to have high application potential in spintronics, energy conversion, highly efficient catalysis, and detection of ambient gas molecules [13,14,15,16,17,18,19,20]. Furthermore, MoSiN has excellent piezoelectric properties, photocatalytic properties, excellent heat transport properties, and application prospects in valley electronics [21,22,23,24,25,26,27]. Bilayer MoSiN and WSiN can change from an indirect to a direct band gap semiconductor under strain and transition from a semiconductor to a metal under external electric field. These characteristics offer basic functions for their application in optoelectronics and electromechanical devices [28,29]. In addition, interesting phenomena can be observed in their heterojunctions. For instance, the van der Waals heterostructures (vdWHs) of MoS_2_/MoSiN and MoSe_2_/MSiN can improve the light absorption capacity [30,31]. The carrier mobility in the latter structure may be an order of magnitude higher than that in the monolayer MoSe_2_. Transformation between type-I and type-II vdWHs and between direct and indirect semiconductors can be realized under strain and external electric fields. The insertion of a graphene layer in the MoGe_2_N_4_/MoSiN heterojunction can greatly reduce the Schottky barrier at the interface and improve the charge injection efficiency [32].

One-dimensional (1D) materials have also attracted attention for high-performance devices due to the expected reduction of electronic scattering phases [33,34]. One-dimensional nanoribbons can be tailored from 2D materials in a top-down way or can be fabricated by assembling atoms precisely in a bottom-up way [35]. Their properties are determined in many cases by edge structures and may differ greatly from those of their 2D counterpart [3,36]. Cutting the monolayer MoSiN into one-dimensional (1D) nanoribbons of different widths and edge configurations (armchair or zigzag) can further modulate the properties. It has been shown that magnetism can be introduced in zigzag MoSiN nanoribbons [37]. In this paper, we show that substitutional doping of 3d transition metal (TM) atoms at the edge sites of Mo atoms in armchair MoSiN nanoribbons (aMoSiNNRs) may realize primary device functions with high performance. This might offer a promising prospect for integrating diversified electronic and spintronic devices based on the monolayer MoSiN, avoiding the previously mentioned structural mismatch challenge.

## 2. Systems and Methods

**Figure 1:**
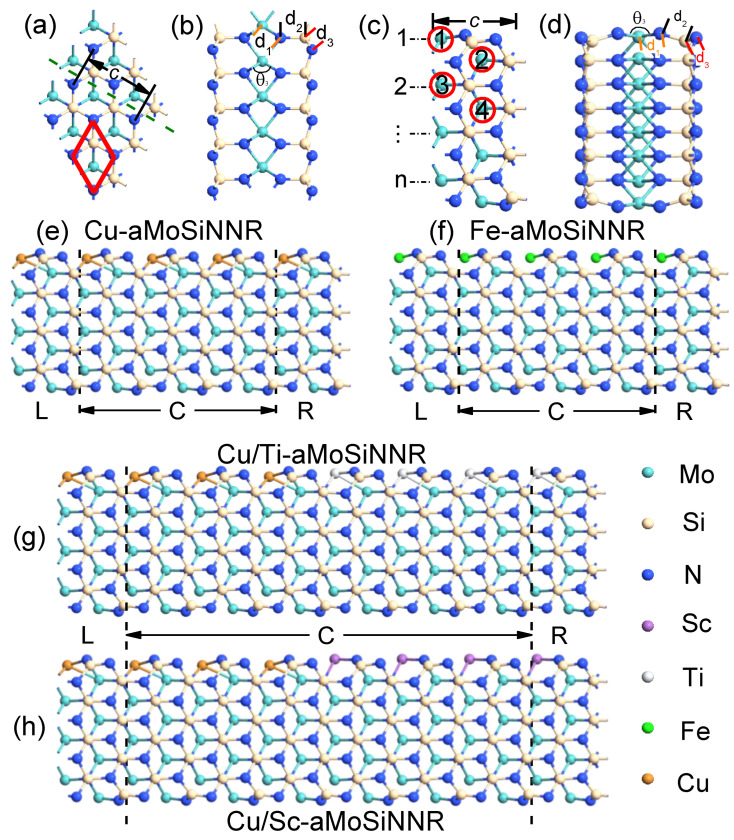
(**a**) Top and (**b**) side views of a 3 × 3 MoSiN supercell. The red rhombus shows a primitive cell, and the olive dashed line indicates the cutting direction for the armchair nanoribbon. (**c**) Top and (**d**) side views of a primitive cell of aMoSiNNR with the width number *n* = 4. The four red circled numbers 1–4 indicate the 4 doping positions discussed. Four typical two-probe devices made of edge-doped aMoSiNNRs, the (**e**) Cu- and (**f**) Fe-doped nanoribbons and the heterojunctions composed of (**g**) Cu/Ti- and (**h**) Cu/Sc-doped nanoribbons. The lattice constant *c* is the period along the ribbon direction for both the 2D and the 1D systems as shown in (**a**) and (**c**), respectively. The bond lengths $d_{1}$, $d_{2}$, and $d_{3}$ plus the angle $\theta_{3}$ for a specific Mo atom in the 2D and 1D systems are marked in (**b**) and (**d**), respectively. Each two-probe system in (**e**–**h**) is partitioned into the left (L) and right (R) electrodes and the central (C) device region. Different atoms are represented by spheres of their own colors as illustrated.

In Figure 1a,b, we show the top and side views of a monolayer MoSiN in a 3 × 3 supercell. The crystal has an atomic septuple-layer structure composed of an N-Mo-N triple layer sandwiched between two Si-N bilayers. It has a hexagonal lattice with the space group P6m1. Along the dashed line in Figure 1a, we can cut the monolayer into aMoSiNNRs of arbitrary width number *n* as shown in Figure 1c,d for the top and side views of a primitive cell at *n* = 4. The lattice constant *c* is defined as the crystal period along the ribbon direction for both the 1D and 2D systems. We will consider the effects of doping by substituting a Mo atom with a TM atom in a supercell. The four doping sites in aMoSiNNR of *n* = 4 are marked by red circles with index numbers in Figure 1c. To facilitate the structural comparison before and after the doping of a TM atom, we define the bond lengths $d_{1}$, $d_{2}$, and $d_{3}$ for the bonds TM-N, N-Si, and Si-N, respectively, along the dashed line direction from the specified TM atom as shown in Figure 1a for MoSiN and in Figure 1c for edge-doped aMoSiNNR. The N-TM-N bond angle θ in the central triple layer is also defined.

Electronic and spintronic devices of high performance may be made from TM-doped MoSiN nanoribbons (TM-aMoSiNNRs). We consider four typical two-probe device systems for the electron transport simulation. The two homojunctions of Cu- and Fe-doped aMoSiNNRs are shown in Figure 1e,f, respectively. The two heterojunctions made from Cu/Ti-doped and Cu/Sc-doped aMoSiNNRs are illustrated in Figure 1g,h, respectively. Each two-probe system is partitioned into three regions, the left (L) and right (R) semi-infinite electrodes and the central region (C), and is connected to a circuit with left (right) Fermi energy $\mu_{L}$ ($\mu_{R}$).

All the simulations are performed by the Atomistix toolkits (ATK) package based on density functional theory [38,39]. The local spin density approximation with the Perdew–Zunger parameterization (LSDA-PZ) is adopted for the exchange–correlation function, and the basis set of double zeta-polarized (dzp) atomic orbits is used. Before calculating the electronic structures and transport properties, the structures are geometrically optimized until the force on any atom is less than 0.02 eV/Å. To avoid any interaction between the adjacent periodic images, vacuum regions at least 20 Å along the directions perpendicular to the 2D monolayer or the 1D nanoribbon are added in the supercell. The cutoff energy is set as 3400 eV. For the 2D monolayer, the *k*-space mesh grid is set to be 10 × 10 × 1, while for the 1D nanoribbon, the k-space mesh grid is set to be 1 × 1 × 101. In the simulation, the electronic temperature of 300 K is assumed in the real axis integration for the non-equilibrium Green’s functions and in the electrodes for transport simulation.

In the transport simulation of the two-probe systems, the current $I_{\sigma }$ of spin σ (↑ or ↓) is evaluated by the Landauer–Büttiker formalism when a voltage bias $V_{b}$ is applied between electrodes L and R [40]:

(1) $$I_{\sigma }=\frac{e}{h}\int_{-\infty }^{\infty }T_{\sigma }\left(E,V_{b}\right)\left[f\left(E-\mu_{L}\right)-f\left(E-\mu_{R}\right)\right]dE$$

Here, $\mu_{L}=-eV_{b}/2$ and $\mu_{R}=eV_{b}/2$ represent the Fermi energies of electrodes L and R, respectively, $T_{\sigma }\left(E,V_{b}\right)$ is the spin-dependent transmission coefficient, and *f* is the Fermi–Dirac distribution function in the electrodes. The current is mainly determined by the transmission coefficient in the transport window $\left[\mu_{L},\mu_{R}\right]$. At zero temperature, the total conductance at zero bias is expressed as $G_{t}=\sum_{\sigma }G_{\sigma }=\sum_{\sigma }e^{2}T_{\sigma }\left(\mu ,0\right)/h$ with $\mu =\mu_{L}=\mu_{R}$.

In the discussion, we define the spin polarization ratio (*SPR*) about the density of states (DOS) at the Fermi energy μ as

$$SPR=\frac{D_{↑}\left(\mu \right)-D_{↓}(\mu )}{D_{↑}(\mu )+D_{↓}(\mu )}$$

with $D_{↑}$ and $D_{↓}$ as the spin-up (↑) and spin-down (↓) DOS, respectively [41]. The spin filtering efficiency (*SFE*) is defined to describe the spin polarization in the conductance and the current at $V_{b}$ as

$$SFE=\frac{I_{↑}(V_{b})-I_{↓}(V_{b})}{I_{↑}(V_{b})+I_{↓}(V_{b})}$$

The diode behavior is described by the rectification ratio (*RR*) defined as $RR=I_{t}(V_{b})/I_{t}(-V_{b})$ with $I_{t}=\sum_{\sigma }I_{\sigma }$ as the total current.

## 3. Results and Discussions

### 3.1. Geometric and Electronic Structures

#### 3.1.1. Pristine Monolayer and Nanoribbon

**Figure 2:**
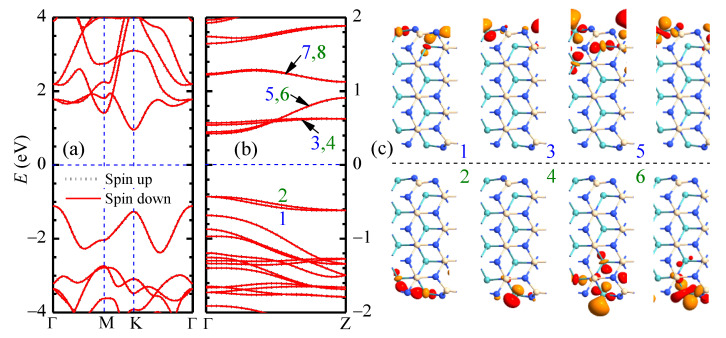
Energy bands of pristine (**a**) MoSiN monolayer and (**b**) aMoSiNNR of *n* = 4 are presented. The Z-point wave functions of bands 1–8 near the Fermi level μ=0 for aMoSiNNR are illustrated in (**c**).

The optimized structure of pristine MoSiN is in agreement with Ref. [29]. The lattice constants are a=b=2.89 Å and the thickness t=6.99 Å. The key structural parameters read $d_{1}=2.10$ Å, $d_{2}=1.73$ Å, $d_{3}=1.74$ Å, and $\theta =74.8^{{}^{\circ}}$. Other parameters include the two N-Si-N angles of $112.3^{{}^{\circ}}$ and $106.5^{{}^{\circ}}$, and the other N-Mo-N angle of $87.0^{{}^{\circ}}$. The energy band of pristine MoSiN in Figure 2a indicates that it is a nonmagnetic indirect semiconductor of energy gap 2.07 eV with the valence band maximum (VBM) at point Γ and the conduction band minimum (CBM) at point K in the first Brillouin zone, in good agreement with the experimental value of 1.94 eV [10]. The obtained DOS/PDOS spectra also agree well with those in Refs. [23,27].

When an aMoSiNNR is cut out from its 2D counterpart, as shown in Figure 1c,d for *n* = 4, obvious structural deformation is observed on the edge due to the emerged dangling bonds. The outer Si-N layer bends inward on the edge, while the edge N atoms beside the edge Mo atom shift slightly outside. The N-Si bond perpendicular to the 2D plane tilts and the typical bond lengths corresponding to site 1, $d_{1}$, $d_{2}$, and $d_{3}$ shrink with a wider angle *θ*, as illustrated in Table 1. The deformation is confined mainly on edge atoms, and the typical structural parameters corresponding to sites 2, 3, and 4 remain almost the same as those in 2D MoSiN. In the following, aMoSiNNRs of only *n* = 4 are considered, and site 1 on the upper edge is the dopant site if not specified.

The confined electron gas in the aMoSiNNR has a much narrower energy gap of 0.854 eV compared to electrons in its 2D counterpart as shown in Figure 2b. This occurs because energy bands of edge states emerge inside the bulk band gap at the Fermi energy. The Z-point wave functions of eight edge bands with a *d* orbital component from the edge Mo atom at site 1 are shown in Figure 2c. States with odd band indices are on the upper edge, and those with even indices are on the lower edge. The states of bands 1 and 2 are composed of Mo *d* orbitals at sites 1 and 2. States 3 and 4 concentrate mainly on the Mo atom at site 1, with tiny components from Si and N atoms around. States 5 and 6 concentrate on site 1 with fractional *d* orbitals at sites 2 and 3. States 7 and 8 are site 1 *d* orbitals with some contribution from its neighboring N atom. Since the bands near the Fermi energy are strongly relevant to the electronic orbitals of the Mo atom at site 1, we can expect a strong property modulation by substitutional doping at site 1.

#### 3.1.2. Nanoribbons Doped by 3d TM Atoms on the Edge

In Table 2, we present the structural parameters, formation energy $E_{\mathrm{form}}$, and the magnetic moment M of TM-aMoSiNNR when the Mo atoms at site 1 on the upper edge are replaced by 3d transition metal (Sc-Zn) atoms. Here, the formation energy is defined as $E_{\mathrm{form}}=(E_{\mathrm{doped}}+E_{\mathrm{Mo}})-(E_{\mathrm{pristine}}+E_{\mathrm{TM}})$ with $E_{\mathrm{doped}}$ as the energy of TM-aMoSiNNR, $E_{\mathrm{pristine}}$ as the energy of pristine aMoSiNNR, $E_{\mathrm{Mo}}$ as the energy of Mo atom, and $E_{\mathrm{TM}}$ as the energy of TM dopant atom. The TM-N bonds around the TM atom have a longer length $d_{1}$ when the 3*d* orbitals are occupied by only one electron (Sc) or fully occupied (Zn) versus those with 3d orbitals that are half occupied.

**Figure 3:**
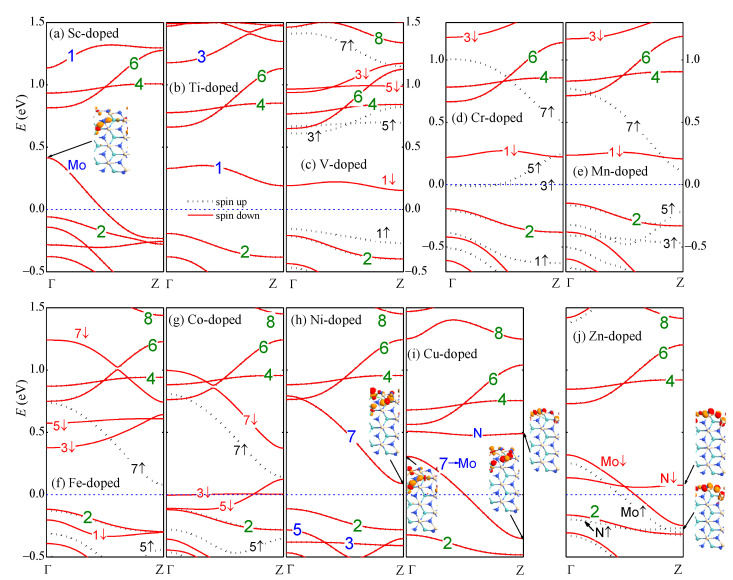
(**a**–**j**) Band structures of 3d TM (Sc-Zn)-doped aMoSiNNRs. Wave functions of some states in typical bands are shown as insets. The Fermi energy is set to zero.

The electronic properties also depend on the atomic number of the edge TM dopant. The formation energy increases oscillatively with the atomic number. The Ti- and V-doped nanoribbons have negative formation energy showing strong stability. The magnetic moment appears maximal ($M=3\mu_{B}$) when the 3*d* orbitals are half occupied (Mn) and reduces an amount of $\mu_{B}$ until zero as the atom number varies for each one. The only exception is the Zn dopant, which has a moment of $\mu_{B}$. The magnetic moment in Zn-aMoSiNNR originates from the spin-polarized N atoms near the Zn dopant. This is different from the other cases where the TM dopants offer the magnetic moment dominantly. In addition, the TM dopant can modulate the conductivity of aMoSiNNR greatly, introducing semiconductor (Ti, Mn, Ni), half-semiconductor (V, Fe), metal (Sc, Cu, Zn), and half-metal (Cr, Co) behaviors as illustrated in the band structures in Figure 3a–j.

**Figure 4:**
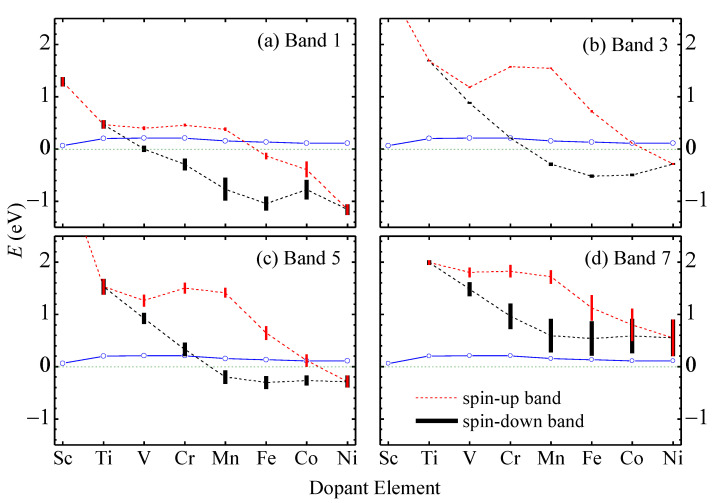
The medium energy (dashed lines) measured from the top of band 2 and the band width (vertical bars) of the spin-up (black) and spin-down (red) branches of (**a**) band 1, (**b**) band 3, (**c**) band 5, and (**d**) band 7 in TM-aMoSiNNRs. The blue empty circles indicate the Fermi energies at low temperature.

With the 3d TM atoms on the upper edge at site 1, bands 2, 4, 6, and 8 of the lower edge states remain almost intact. Bands 1, 3, 5, and 7 of the upper edge states may shift in a large range of energy depending on the atomic number of the dopant, as shown in Figure 4a–d, respectively. In the Sc-doped case, there are three less electrons in the outer valence shell of the TM dopant compared to the host Mo atom. Band 1 moves up beyond the Fermi level and becomes unoccupied. A new band of the upper edge states noted as band Mo in Figure 3a emerges from the bulk valence bands and becomes half occupied. Band Mo is composed of *d* orbitals from the Sc dopant atom and the Mo atoms at sites 2 and 3. The Sc-aMoSiNNR then becomes a metal. In Ti-aMoSiNNR, there is one more electron in each primitive cell. The above emerged band in the Sc-doped nanoribbon becomes fully occupied again and sinks deep into the bulk bands. The nanoribbon becomes a semiconductor with an indirect band gap of 0.382 eV as shown in Figure 3b. In V-aMoSiNNR, with one more electron further, the last electron goes to band 1 since it is just above band 2. Different from the previous two doped cases, spin split occurs here, and only the spin-up branch of band 1 is occupied. The nanoribbon becomes a spin-down half-semiconductor with a direct spin-up band gap of 0.765 eV and an indirect spin-down band gap of 0.362 eV as illustrated in Figure 3c. The Cr dopant atom has a magnetic moment of $2\mu_{B}$, and the Zeeman split of the upper edge bands in Cr-aMoSiNNR becomes much bigger. The spin-up branches of bands 3 and 5 move below the spin-down branches of band 1, and the added electron occupies half in these two spin-up branches. The nanoribbon becomes a spin-up half-metal as in Figure 3d. The Mn dopant has the maximum magnetic moment of $3\mu_{B}$, and the spin-up branches of the upper edge bands 1, 3, 5 are all occupied. The Mn-aMoSiNNR becomes a magnetic semiconductor with a band gap of about 0.35 eV for both spin-up and -down electrons as presented in Figure 3e. Here, the energy of the ferromagnetic structure ($E_{FM}$) is 16.5 meV lower than the energy of the antiferromagnetic structure ($E_{AFM}$). From the expression $k_{B}T_{C}\approx 2(E_{AFM}-E_{FM})/3$ in the Heisenberg’s mean field approximation, we obtain the Curie temperature $T_{C}\approx 128$ K for Mn-aMoSiNNR [42]. In Fe-aMoSiNNR, the spin-down branch of band 1 is now occupied, but the corresponding spin-up branch is located deep inside the bands of the bulk states. The nanoribbon becomes a spin-up half-semiconductor with band gaps of 0.209 and 0.494 eV for spin-up and spin-down electrons, respectively. The Co-aMoSiNNR is a spin-down half-metal since spin-down branches of bands 3 and 5 are both half occupied, as shown in Figure 3g.

When the dopant atomic number increases, states in band 7 receive increasing components of Mo orbitals at sites 2 and 3. Accordingly, band 7 becomes more dispersed when shifting down with bands 1, 3, and 5, as illustrated in Figure 3c–d. In Ni-aMoSiNNR, which is an indirect semiconductor of band gap 0.202 eV, the components from sites 2 and 3 are comparable with that from site 1, as shown in the inset of Figure 3h. In Cu-aMoSiNNR, they become dominant components, and the wave functions of states in band 7 are similar to those in band Mo of Sc-aMoSiNNR. Here, band Mo is also half occupied and Cu-aMoSiNNR is also a metal as in the Sc-doped case. At the same time, another band, noted as band N, whose states are composed of orbitals from edge N atoms near the dopant, emerges just below bands 4 and 6 as shown in Figure 3i. In Zn-aMoSiNNR, band N splits into two spin branches and introduces a magnetic moment of $\mu_{B}$ since only the spin-up branch is occupied.

In Figure 4, we plot the medium band energies (dashed) measured from the top of band 2 and the band widths (vertical bar) of upper edge bands 1, 3, 5, and 7 versus the dopant atomic number for elements from Sc to Ni. The Fermi level (solid) is pinned within 0.2 eV above the top of band 2. The upper edge bands shift downward relative to the lower edge bands, as more electrons exist in the upper edge. The spin split of the upper edge bands is approximately proportional to the magnetic moment of the dopant atom. Band 3 is always very narrow since the corresponding states are basically localized on the dopant atom. Band 5 has a steady dispersion due to the component from Mo orbitals at site 2 in the corresponding states. Band 1 becomes wider when located below the Fermi energy where coupling between its states and other states occurs. Band 7 becomes wider and wider as its energy lowers, and its states contain higher and higher fractions of orbitals at sites 2 and 3. In Cu- and Zn-doped nanoribbons, the upper edge bands are difficult to identify and are not shown, since they are deep below the Fermi level, and the corresponding states are mixed with states in other bands. Note that Cr has a valence configuration of 3d^5^4s^1^ similar to that of the host element Mo 4d^5^5s^1^, but the Cr-doped nanoribbon shows quite different electronic band structure and physical properties from the pristine nanoribbon.

**Figure 5:**
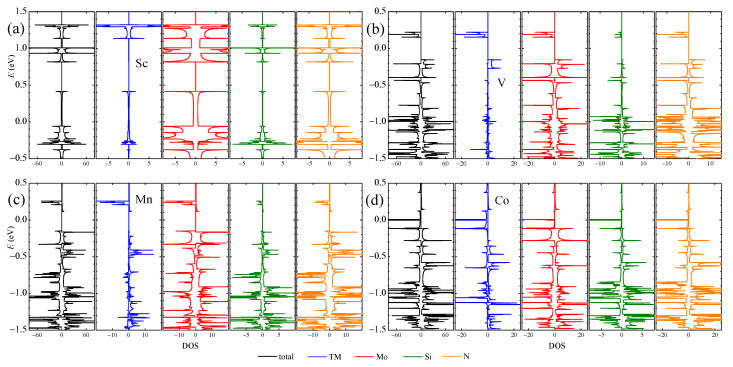
DOS/PDOS of (**a**) Sc-, (**b**) V-, (**c**) Mn- and (**d**) Co-doped aMoSiNNRs. The spin-up and spin-down components are plotted on the positive and negative sides, respectively.

The DOS and partial DOS (PDOS) from different elements in four typical TM-aMoSiNNRs are shown in Figure 5. The DOS and PDOSs of Sc-doped nanoribbons are spin-up and -down symmetric, indicating no magnetic signal. The DOS at the Fermi level is dominantly from the Mo element, confirming the contribution of the Mo band in Figure 3a. In the V-aMoSiNNR, the DOS and PDOS are asymmetric, and the resultant magnetic moment originates from band 1 with contributions mainly from elements V and Mo. In the Mn-aMoSiNNR, the magnetic moment comes mainly from the spin-up electrons of about −0.5 eV where bands 3 and 5 are located and are around −1.3 eV where band 1 lies. In Co-aMoSiNNR, the DOS at the Fermi energy diverges and is 100% spin-down polarized since both spin-down branches of bands 3 and 5 cross the Fermi level. The magnetic moment comes mainly from the dopant atom.

#### 3.1.3. Nanoribbons Doped by 3d TM Atoms Inside

**Figure 6:**
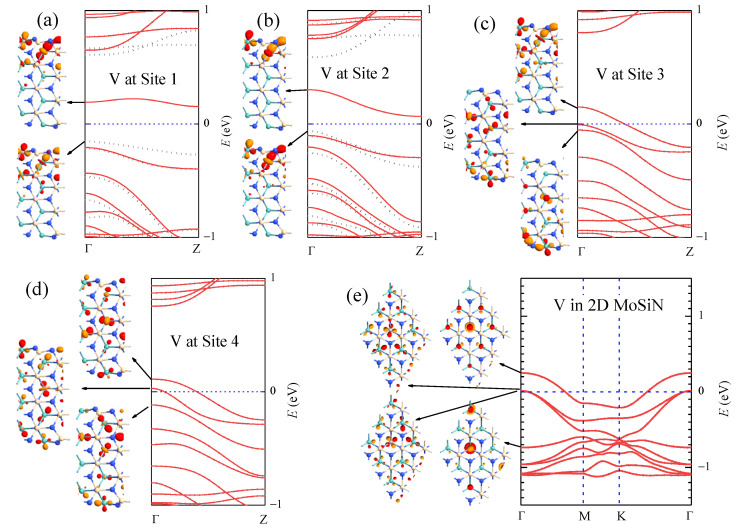
Band structure of aMoSiNNR doped by V atoms at (**a**) site 1, (**b**) site 2, (**c**) site 3, and (**d**) site 4. The band structure of the 2D MoSiN doped by one V atom in each 3 × 3 supercell is shown in (**e**). The Γ point wave functions of states in some bands near the Fermi level are plotted as insets.

In Table 3, we list the formation energy $E_{f}$ and the magnetic moment M, together with the structural parameters *c*, $d_{1}$, $d_{2}$, $d_{3}$, and *θ* associated with the dopant sites when V atoms substitute Mo atoms at sites 1–4 in aMoSiNNRs. The parameters for the V dopant at site 4 is quite close to those for the V dopant in 2D MoSiN, indicating that the effect of the V dopant in the center of the nanoribbons is similar to that in MoSiN. This is confirmed by the corresponding band structures and wave functions as illustrated in Figure 6. In nanoribbons doped by V at site 1, as shown in Figure 6a and in previous discussions, the magnetic moment comes from the spin split of band 1, whose states are composed of *d* orbitals from TM atoms at sites 1 and 2. When the V dopant moves from site 1 to site 2, the energy and the wave functions of the states in band 1 do not change much as illustrated in Figure 6b. The band structure varies only slightly with the minimal spin split of band 1 narrowed from 0.31 to 0.13 eV, and the magnetic moment ($\approx \mu_{B}$) remained almost the same. If the V dopant is located at site 3, a band of bulk states composed of mainly V and Mo *d* orbitals emerges up to the Fermi level, and band 1 shifts upward and away from band 2. At the same time, states in bands 1 and 2 mix with the bulk states and become more extensive, as shown in the inset of Figure 6c. The spin split disappears, and band 1 becomes half occupied, making the nanoribbon a metal. In the system of the V dopant at site 4, the edge components of the states in the bands near the Fermi energy become minimal. States in band 1 are composed of mainly the dopant orbitals at site 4 on the upper side and the Mo orbitals at site 4 on the lower side. The emerged bulk band crosses through the Fermi level and becomes partially occupied as shown in Figure 6d. In the last case, the situation becomes similar to that in V-doped 2D MoSiN, where the states in the conduction bands are composed of orbitals of the dopant V atom and of the Mo atoms around the V atom, as shown in Figure 6e for a system with the 3 × 3 supercell. Similarly, when moving the dopant site from the edge to the center in aMoSiNNRs doped by other 3*d* TM elements (Sc, Ti, and Cr-Zn), doping characteristics gradually approach those observed in 2D MoSiN doped by the same element.

### 3.2. Electron Transport

**Figure 7:**
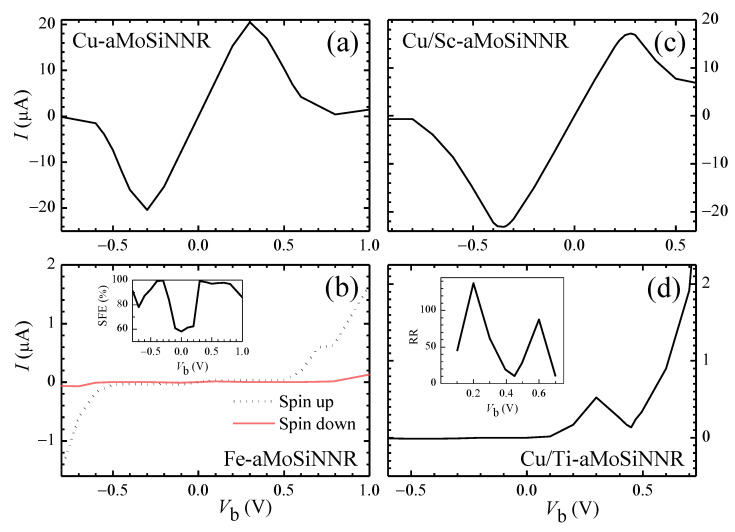
The *I-V* characteristics of two-probe device systems made of aMoSiNNR homojunctions doped by (**a**) Cu and (**b**) Fe elements, and heterojunctions doped by (**c**) Cu/Sc and (**d**) Cu/Ti elements at site 1. The spin filtering efficiency of the Fe homojunction and the rectification ratio of the Cu/Ti heterojunction are plotted as the insets of (**b**) and (**d**), respectively.

As discussed above, aMoSiNNRs doped by 3d TM atoms can exhibit a wealth of physical properties. Pristine nanoribbons can be converted by edge doping from a nonmagnetic direct semiconductor into a nonmagnetic indirect semiconductor (Ti, Ni), magnetic semiconductor (Mn, Fe, V), spin-up half semiconductor (Fe), spin-down half semiconductor (V), nonmagnetic metal (Sc, Cu), magnetic metal (Zn, Cr, Co), spin-up half metal (Cr), and spin-down half metal (Co). The magnetic moment per dopant can vary from 0 to 3$\mu_{B}$. These properties are beneficial to the development of electronic and spintronic devices. The substitutional doping and the heterojunction structures, which have been widely employed in devices, can further modify the device performance of the materials. In Figure 7a–d, we present the current–voltage (*I–V*) characteristics and performances of the four typical two-probe devices schemed in Figure 1e–h, respectively.

In Figure 7a a single-band *I–V* characteristic with strong negative differential resistance is observed for the Cu edge-doped homojunction. The peak-to-valley ratio of the *I-V* curve may reach as high as 142. This high device performance originates from the corresponding band structure and orthogonality between states in conduction and valence bands as shown in Figure 3i. The conduction band is band Mo of states mainly composed of Mo orbitals at sites 2 and 3 on the upper side. A band gap exists above the conduction band, and the states in the conduction band match badly with those in the valence bands. The conduction band becomes the only band contributing to the electron transport in the considered range of voltage bias. The current reaches a minimum near $V_{b}$ = 0.7 V when the Fermi level difference between the two electrodes is around the band width of the conduction band [43].

The *I–V* behavior of the spin-up half semiconductor Fe-aMoSiNNR is shown in Figure 7b. The system works as an insulator for spin-down electrons but as a semiconductor for spin-up electrons. The spin filtering efficiency is close to or higher than 80% when the device is on. Different from the pure spin filtering system of half metals such as Cr- and Co-aMoSiNNRs, here, a nonlinear *I–V* curve is observed, and the current can be greatly modulated by the voltage bias.

In Figure 3a,i it is shown that the conduction band of Sc-aMoSiNNR and Cu- aMoSiNNR is the same band called Mo. This suggests that the two nanoribbons may match well in view of electron transport. In Figure 7c, we present the *I–V* curve of the Cu/Sc-aMoSiNNR heterojunction schemed in Figure 1g. The *I–V* curve is quite similar to that of the Cu-aMoSiNNR homojunction as shown in Figure 7a with strong negative differential resistance. High diode performance can also be realized in TM-doped aMoSiNNR systems as shown in Figure 7d for Cu/Ti heterojunction. A positive (negative) $V_{b}$ can switch on (off) the junction with a rectification ratio up to RR = 137 at $V_{b}$ = 0.2 V.

**Figure 8:**
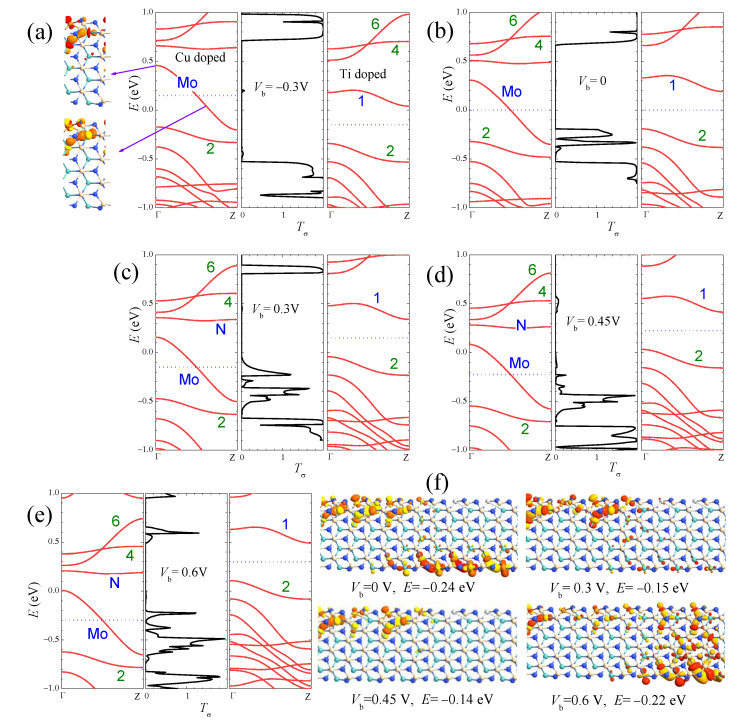
The combination plots of the band structure for left electrode (left panels), the transmission spectra $T_{\sigma }$ (middle panels), and the band structure for the right electrode (right panels) at the bias of (**a**) −0.3 V, (**b**) 0, (**c**) 0.3 V, (**d**) 0.45 V, (**e**) 0.6 V for the Cu/Ti-aMoSiNNR heterojunction. The transmission eigenstates at some typical values of $V_{b}$ and *E* are shown in (**f**).

The mechanism of the *I–V* curves on different devices can be well understood by the bias-dependent transmission spectra and the matching of band structures and wave functions in the electrodes. As an example, in Figure 8, we present the explanation of the *I–V* curve for the Cu/Ti heterojunction by the combination plots of band structures and wave functions in the two electrodes and the transmission spectra. As discussed in Equation (1), the current *I* can be approximately estimated by integrating the transmission coefficient (*T*) over the energy range of the transport window $\left[\mu_{L}=-eV_{b}/2,\mu_{R}=eV_{b}/2\right]$. Because the distribution functions *f* of the left and right electrodes differ greatly only for states with energies inside the transport window, electrons in these states may contribute to a net current *I*. In Figure 8, the transport windows are indicated by the blue dotted lines. The size and position of the transmission peaks vary with the voltage bias. Under negative biases such as $V_{b}$= −0.3 V in Figure 8a, the energy bands in the left (right) electrodes shift up (down) by $\left|eV_{b}/2\right|$. Inside the transport window, electrons in only a narrow range of energies from the left electrode can transfer to the overlapping band 1 in the right electrode. However, due to the wave function mismatch between the states in band Mo of Cu-aMoSiNNR and the states in band 1 of Ti-aMoSiNNR, only a tiny transmission peak appears at *E* = 0.2 eV where a divergent DOS exists at band 1 in the right electrode. The contribution of the tiny transmission peak to the current is negligible, and the Cu/Ti heterojunction under negative bias is off. Under positive bias $V_{b}>0$, band 2 and some bulk bands below enter the transport windows as $V_{b}$ increases and contribute to the current as shown in Figure 8b–e. At low bias $V_{b}=0$, the sharp transmission peak at *E* = −0.25 (−0.33) eV is due to the wave function match between left band Mo (band 2) and right band 2. With the increase in $V_{b}$ and the widening of the transport window, the two peaks approach and enter into the transport windows but at the same time diminish gradually due to the mismatch of wave functions and bands. The competition of these two factors results in a current maximum at $V_{b}$ = 0.3 V and a minimum at $V_{b}$ = 0.45 V. Accordingly a negative differential resistance appears between 0.3 and 0.45 V with a peak-to-valley ratio of 4. The variation of transmission coefficient with the bias is also illustrated by the transmission eigenstates in Figure 8f. The transport channel from left band Mo to right band 2 is open at $V_{b}=0$ as shown by the transmission eigenstate at *E* = −0.24 eV. It becomes partially closed when it enters into the transport window at $V_{b}$ = 0.3 V, as shown by the transmission eigenstate at *E* = −0.15 eV, and is completely closed at $V_{b}$ = 0.45 V. Under higher bias $V_{b}$ = 0.6 V, as shown by the transmission eigenstate at *E* = −0.22 eV, the bulk bands in the right electrode enter into the transport window and contribute significantly to the current.

## 4. Conclusions

We systematically studied the geometries, electronic band structures, and electron transport properties of armchair MoSi_2_N_4_ nanoribbons when the Mo atoms are substituted by 3d TM atoms at some crystal sites (TM-aMoSiNNRs). The density functional theory combined with the non-equilibrium Green’s function method is employed in the simulations. The doping may significantly deform the structure on the edge of the nanoribbons but little affects the structure in the center. This may introduce doping effects in nanoribbons distinguished from those in their 2D counterparts. In all the cases, the lattice constant remains basically the same; thus, perfect structural matching can be easily realized in combining different TM-aMoSiNNRs into integrated circuits. Nevertheless, band structures near the Fermi energy can be greatly modulated, and magnetism can be introduced with a moment up to $3\mu_{B}$ in Mn-aMoSiNNR. The pristine nonmagnetic direct semiconductor aMoSiNNR can then be converted by doping into a nonmagnetic indirect semiconductor (Ti, Ni), magnetic semiconductor (Mn, Fe, V), spin-up half semiconductor (Fe), spin-down half semiconductor (V), nonmagnetic metal (Sc, Cu), magnetic metal (Zn, Cr, Co), spin-up half metal (Cr), and spin-down half metal (Co) to realize different device functionalities. Cr- and Co-aMoSiNNRs can be used as perfect spin-up and spin-down filtering valves, respectively, and Fe- and V-aMoSiNNRs as single spin semiconductors. Both the Cu-aMoSiNNRs homojunction and the Cu/Sc-aMoSiNNR heterojunction can be used to make single-band negative differential resistance devices with high peak-to-valley ratios. The Cu/Ti-aMoSiNNR heterojunction can be employed for a high-quality diode. These colorful properties of structurally matched aMoSiNNRs functionalized by 3d TM elements can greatly broaden their potential application in integrated electronic and spintronic devices. Note that our simulations of the electronic behaviors are carried out with simplified models under ideal conditions. Many-body effects and random scatterings with impurities, edge roughness, and phonons are not fully taken into account. The described phenomena might be quantitatively modified in realistic systems.

## Figures and Tables

**Table 1:** The typical structural parameters *c*, $d_{1}$, $d_{2}$, $d_{3}$, and *θ* associated with sites 1–4 in pristine aMoSiNNR of *n* = 4 are compared with those in 2D MoSiN.

Config.	*c* (Å)	*d*_1_ (Å)	*d*_2_ (Å)	*d*_3_ (Å)	*θ* (°)
Site 1	4.99	1.97	1.67	1.62	80.5
Site 2	4.99	2.09	1.78	1.73	74.5
Site 3	4.99	2.10	1.75	1.73	73.7
Site 4	4.99	2.10	1.73	1.73	73.9
MoSiN	4.99	2.10	1.73	1.74	74.8

**Table 2:** The typical structural parameters *c*, $d_{1}$, $d_{2}$, $d_{3}$, and *θ* associated with site 1, the formation energy $E_{f}$, and the magnetic moment M of aMoSiNNRs undoped and doped by 3*d* TM elements (Sc-Zn) at site 1.

Dopant	*c* (Å)	*d*_1_ (Å)	*d*_2_ (Å)	*d*_3_ (Å)	*θ* (°)	$E_{f}$ (eV)	M (*μ_B_*)
Pristine	4.99	1.97	1.67	1.62	80.5		0.00
Sc	4.99	2.06	1.65	1.62	74.9	0.376	0.00
Ti	4.99	1.90	1.66	1.61	85.6	−1.491	0.00
V	4.99	1.84	1.67	1.61	87.8	−1.013	1.00
Cr	4.99	1.85	1.67	1.61	85.8	1.219	2.00
Mn	4.99	1.86	1.66	1.61	84.3	2.474	3.00
Fe	4.99	1.85	1.67	1.62	82.6	1.506	2.01
Co	4.99	1.86	1.67	1.62	81.6	1.534	1.00
Ni	4.99	1.89	1.66	1.62	80.5	1.752	0.00
Cu	4.99	1.93	1.67	1.62	78.6	5.126	0.00
Zn	4.99	2.01	1.66	1.62	78.2	7.722	1.08

**Table 3:** The typical structural parameters *c*, $d_{1}$, $d_{2}$, $d_{3}$, and *θ*, the formation energy $E_{f}$, and the magnetic moment M of V-aMoSiNNRs for dopant sites 1–4 and of V-doped MoSiN.

Dopant Environment	*c* (Å)	*d*_1_ (Å)	*d*_2_ (Å)	*d*_3_ (Å)	*θ* (°)	$E_{f}$ (eV)	M (*μ_B_*)
V-site1	4.99	1.84	1.67	1.61	87.8	−1.013	1.00
V-site2	4.98	2.02	1.78	1.73	74.6	−0.260	0.97
V-site3	4.97	2.03	1.75	1.73	75.1	−0.302	0.00
V-site4	4.98	2.01	1.73	1.73	75.1	−0.171	0.00
V-MoSiN	4.99	2.02	1.73	1.74	75.1	−0.165	0.00

## Data Availability

Data is contained within the article.
